# lncRNA PCBP1-AS1 mediated downregulation of ITGAL as a prognostic biomarker in lung adenocarcinoma

**DOI:** 10.18632/aging.204756

**Published:** 2023-05-31

**Authors:** Qiang Wang, GuangJun Xiao, Na Li, Xiulin Jiang, Chunhong Li

**Affiliations:** 1 Gastrointestinal Surgical Unit, Suining Central Hospital, Suining 629000, Sichuan, P.R. China; 2 Department of Clinical Laboratory Medicine, Suining Central Hospital, Suining 629000, Sichuan, P.R. China; 3 Department of Oncology, Suining Central Hospital, Suining 629000, Sichuan, P.R. China

**Keywords:** integrin alpha L, immunotherapy, immune checkpoint inhibitors, NSCLC, lncRNA

## Abstract

Integrin alpha L (ITGAL) seemed to play a critical role in carcinogenesis and immune regulation. Nevertheless, the effects of ITGAL on non-small cell lung cancer (NSCLC) remain elusive. The present paper intended to determine the effects of ITGAL in NSCLC via the integration of bioinformatic analyses. In this study, we found that the mRNA and protein levels of ITGAL were downregulated in NSCLC tissues. Significantly, low ITGAL expression was related to poorer prognosis and increased malignancy of NSCLC. In addition, GO analysis and KEGG pathway analysis revealed that the coexpressed genes of ITGAL were predominantly associated with various immune-associated signaling pathways, like the T cell receptor signaling pathway, Th17 cell differentiation, chemokine signaling pathway, and NF-κB signaling pathway. Our result indicated that lncRNA-mediated downregulation of integrin alpha L expression was tightly related to immunocyte infiltration, immune modulators, and chemotactic factors in NSCLC, which potentially serves as a biomarker for clinical prognosis prediction and immunotherapy of NSCLC. This is the first comprehensive analysis of ITGAL in the prognosis, immune microenvironment, and immunotherapy of lung adenocarcinoma. ITGAL are promising biomarkers for predicting clinical outcomes and immunotherapy responses in patients with NSCLC.

## INTRODUCTION

Lung cancer is a serious cancer that poses a great threat to human health and brings a great burden to society [1]. Unfortunately, due to the lack of effective early diagnostic indicators, most lung cancer patients showed extremely poor prognosis for these patients, with a five-year survival rate of only 4% [2]. Over the past decade, several immune checkpoint inhibitors (ICIs), including ipilimumab, pembrolizumab, and atezolizumab, have been widely used in the treatment of advanced NSCLC. [3–8]. Therefore, it is crucial to discover new prognostic gene signatures that can be used not only to predict patient prognosis but also as new therapeutic targets for lung cancer patients.

ITGAL, also name CD11a, is a differential that existed in diverse immune cells and modulates the intercellular adhesion of lymphocytes [9]. Studies confirmed that ITGAL plays crucial roles in cancer progression and tumor immune microenvironment [10]. Mutation of ITGAL promotes the susceptibility of salmonella enterica to serovar Typhimurium [11]. It has been confirmed that ITGAL is related to poor prognosis and immunity in acute myeloid leukemia [12]. In glioma, results show that ITGAL is up-regulated and knockout ITGAL inhibited CX3CL1-directed motility [13]. However, the potential mechanisms of ITGAL involved in lung cancer malignant progression and immune immunotherapy of NSCLC are still unknown.

Herein, we determine the expression levels, clinical features, prognosis, and diagnostic values of ITGAL in NSCLC by TCGA-NSCLC datasets. Moreover, we evaluated its correlation with tumor immune infiltration in NSCLC by the TISIDB database and single-cell sequencing data. DNA methylation of ITGAL in NSCLC examined by Gene Set Cancer Analysis database.

## MATERIALS AND METHODS

### Data collection and processing

We obtained the transcriptome profiles of 504 LUAD samples and 59 samples from healthy lung tissue from the TCGA database (https://cancergenome.nih.gov/).

### Gene set cancer analysis (GSCA)

GSCA (http://bioinfo.life.hust.edu.cn/GSCA/#/document) is an integrated platform including the RNA levels, DNA methylation, immune cell infiltration, and drug resistance of TCGA pan-cancer [14].

### The cancer therapeutics response portal database

CTRP (http://portals.broadinstitute.org/ctrp/) database is a database, which includes the protein-kinase-targeting drugs and genomic alterations data [15]. In this manuscript, we employed the CTRP to explore the relationships between ITGAL and the sensitivity of different drugs.

### Linkedomics database

Linkedomics is a database, which includes biologists and clinicians across tumor types in TCGA [16]. In this study, we determine the potential biological and signaling pathway of ITGAL in NSCLC by Linkedomics.

### Human protein Atlas database

HPA is a comprehensive TCGA cancer-related database [17]. In this finding, we were using the HPA database analysis of the protein of ITGAL in lung tissues and lung cancer tissues.

### TISIDB database

TISIDB is a tumor immune-related database [18]. In this study, we use TISIDB to examine the correlations between ITGAL and different immune modulators in NSCLC.

### Single-cell sequencing analysis

Single-cell sequencing analysis (http://lung.cancer-pku.cn/index.php). In this study, we examined the correlations between ITGAL and different T cells in NSCLC.

### Kaplan-Meier plotter

In this manuscript, we analysis of the relationship between ITGAL and overall survival and immune cell in patients with NSCLC by Kaplan-Meier Plotter database.

### qRT-PCR assay

The lung cancer cell line was purchased from the ATCC cell bank and cultured using RPMI-1640 medium. Actin as an internal reference gene for qPCR assay. The primer of ITGAL is as follows: qPCR-F: TGCTTATCATCATCACGGATGG, qPCR-R: CTCTCCTTGGTCTGAAAATGCT.

## RESULTS

### ITGAL is decreased in NSCLC

**Figure 1:**
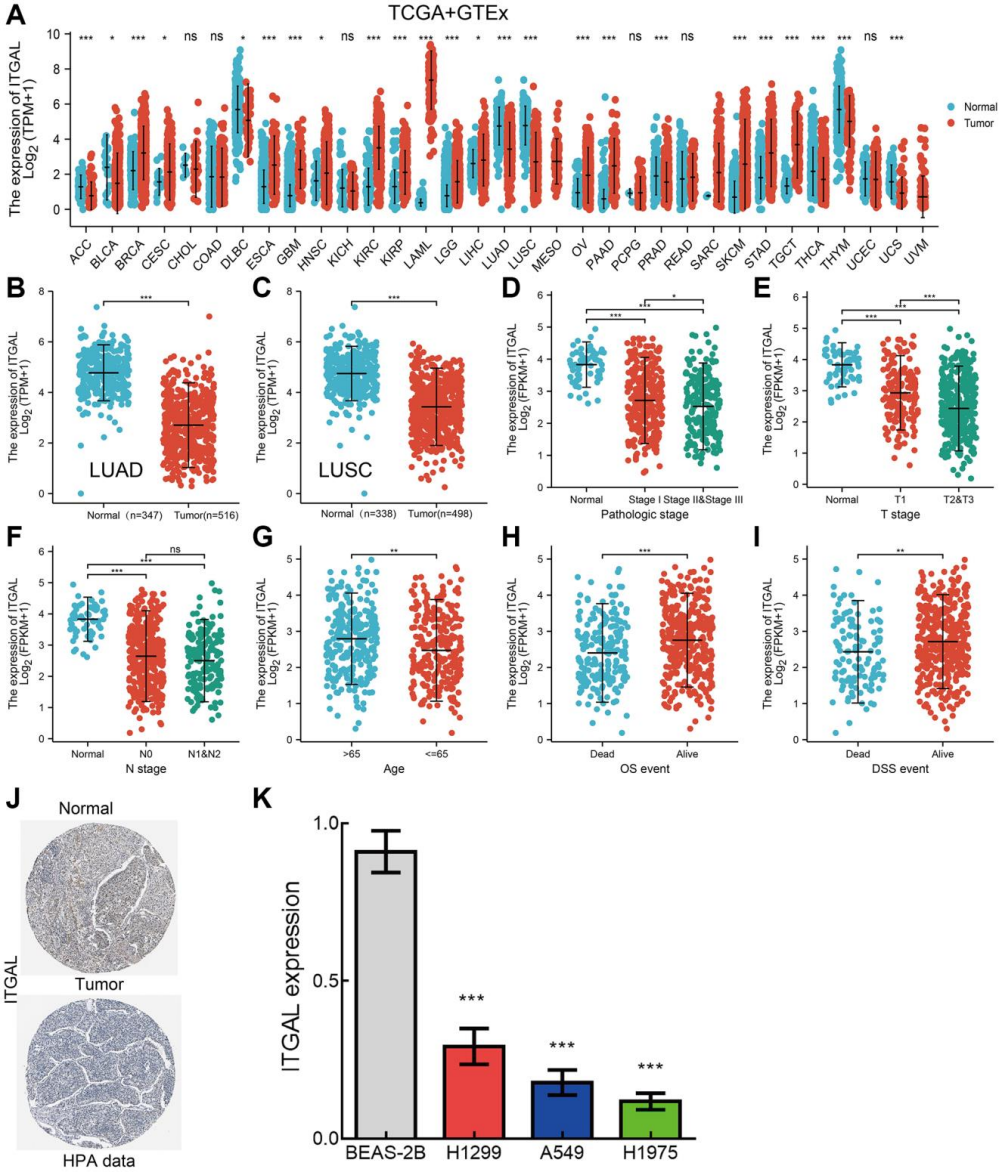
**ITGAL was down-regulated in NSCLC.** (**A**) ITGAL level in diverse cancer types by TCGA database. (**B**, **C**) ITGAL was down-regulated in NSCLC by the TCGA database. (**D**–**I**) Correlation between ITGAL expression and diverse clinical features in NSCLC by TCGA database. (**J**) The protein expression of ITGAL in lung cancer tissues examined by IHC assay. (**K**) The RNA expression of ITGAL in lung cancer cell lines examined by qPCR assay, Actin as an internal reference gene. NS > 0.05, ^*^p < 0.05, ^**^p < 0.01, ^***^p < 0.001.

**Figure 2:**
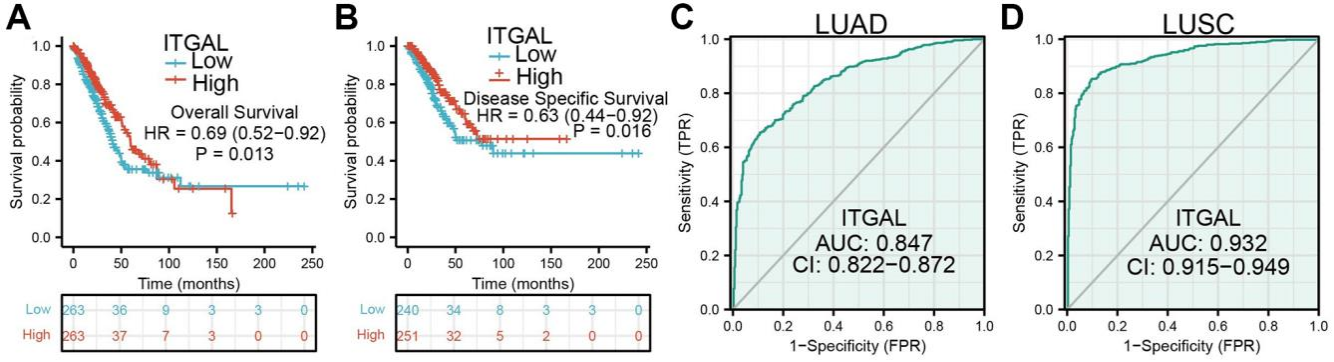
**Prognosis of ITGAL in NSCLC.** (**A**, **B**) OS and DSS of ITGAL in NSCLC. (**C**, **D**) Diagnostic value of ITGAL in NSCLC.

In the TCGA dataset, we found that ITGAL was down-regulated in NSCLC (Figure 1A–1C). We next explored the correlations between ITGAL level and clinical features of NSCLC patients. Results showed that low expression of ITGAL related to poor clinical features, including pathological, TN stage, age, and diverse survival events (Figure 1D–1I). Using the HPA database, we confirmed that ITGAL was significantly down in lung cancer tissue (Figure 1J). More importantly, compared to normal lung epithelial cells, ITGAL levels in lung cancer tumor cell lines are abnormally decreased (Figure 1K). Kaplan-Meier was employed to determine the prognosis of ITGAL in NSCLC. We found that patients with a lower level of ITGAL have short OS and DSS in NSCLC patients (Figure 2A, 2B). ROC curve data showed that ITGAL may be a potential diagnostic marked in NSCLC (Figure 2C, 2D).

### Single-cell sequencing analysis

**Figure 3:**
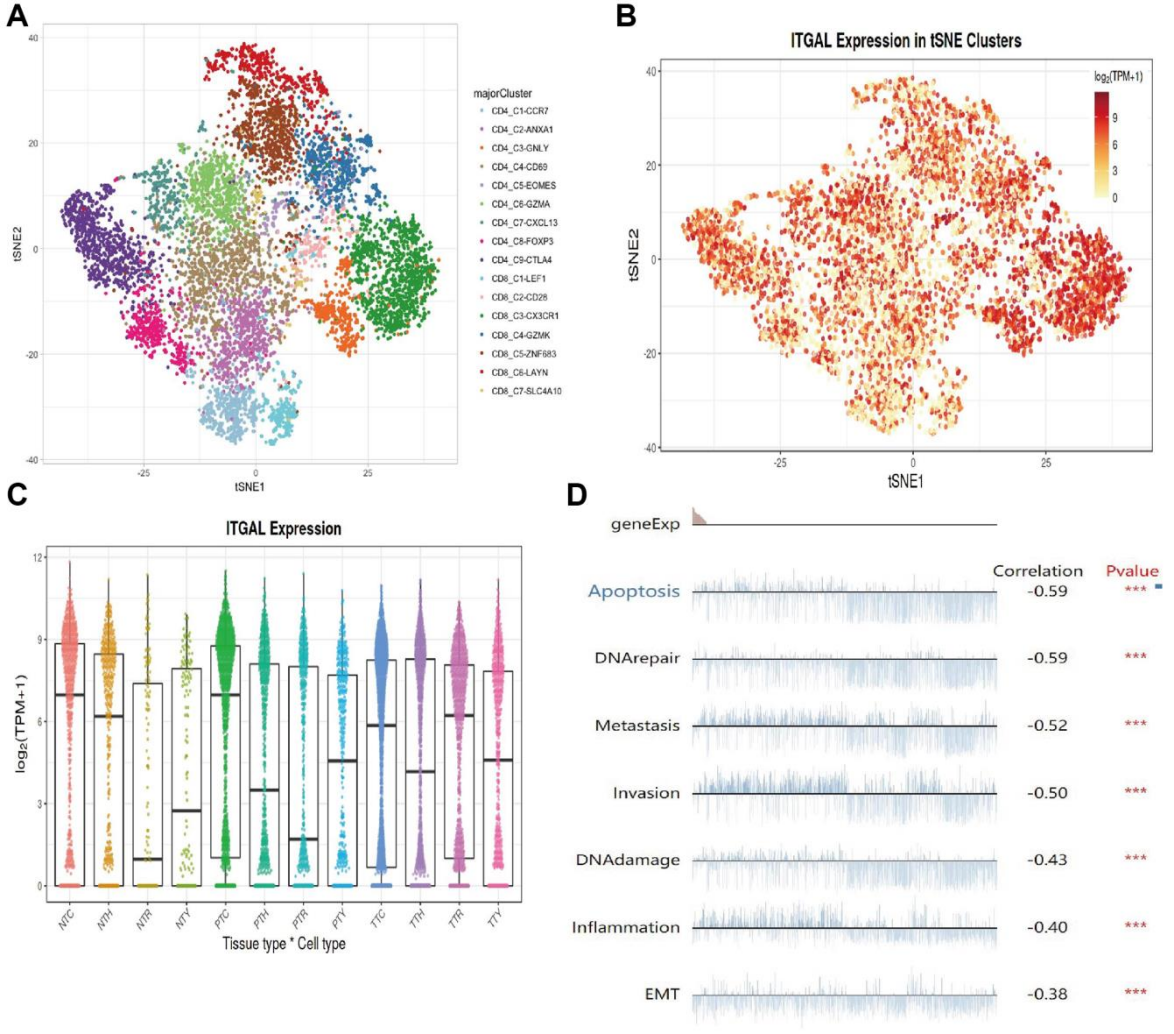
**Single-cell sequencing analysis.** (**A**–**C**) The expression of ITGAL in diverse immune cells. (**D**) Analysis of the function of ITGAL in NSCLC by using single-cell sequencing data. ^***^p < 0.001.

We examined single-cell transcriptome data of NSCLC and found that ITGAL has different expression modes in diverse immune cells of NSCLC (Figure 3A, 3B). ITGAL major is highly expressed in PTC immune cells and downregulated in NTR immune cells (Figure 3C). Single-cell transcriptome data used conducted function analysis. Results demonstrated that ITGAL level was negatively related to cell apoptosis and EMT (Figure 3D).

### KEGG and DNA methylation analysis

**Figure 4:**
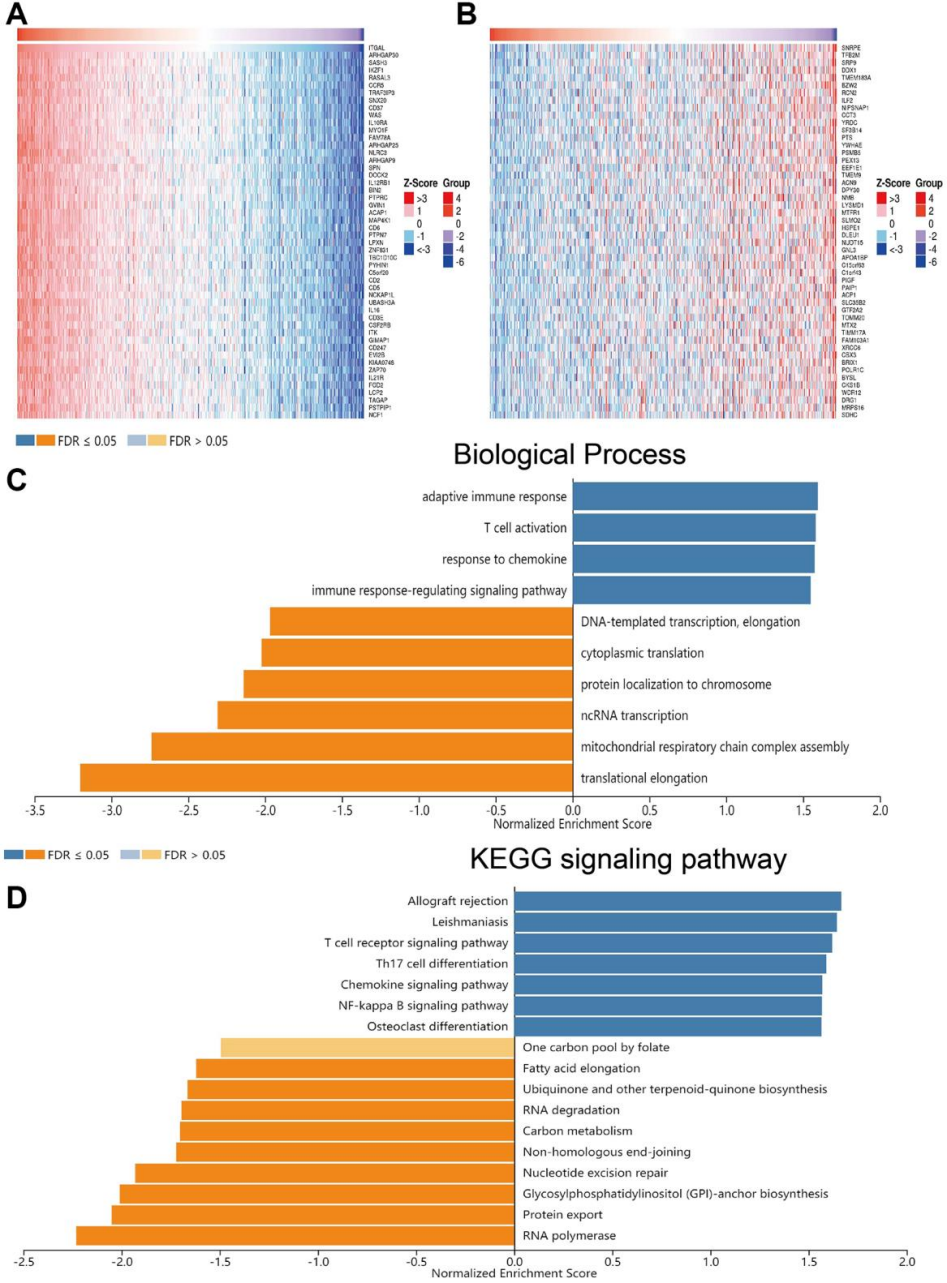
**GO and KEGG enrichment analysis.** (**A**, **B**) Genes that are positive or negative with ITGAL in NSCLC were examined by Linkedomics. (**C**, **D**) GO and KEGG enrichment analysis of ITGAL in NSCLC examined by Linkedomics.

**Figure 5:**
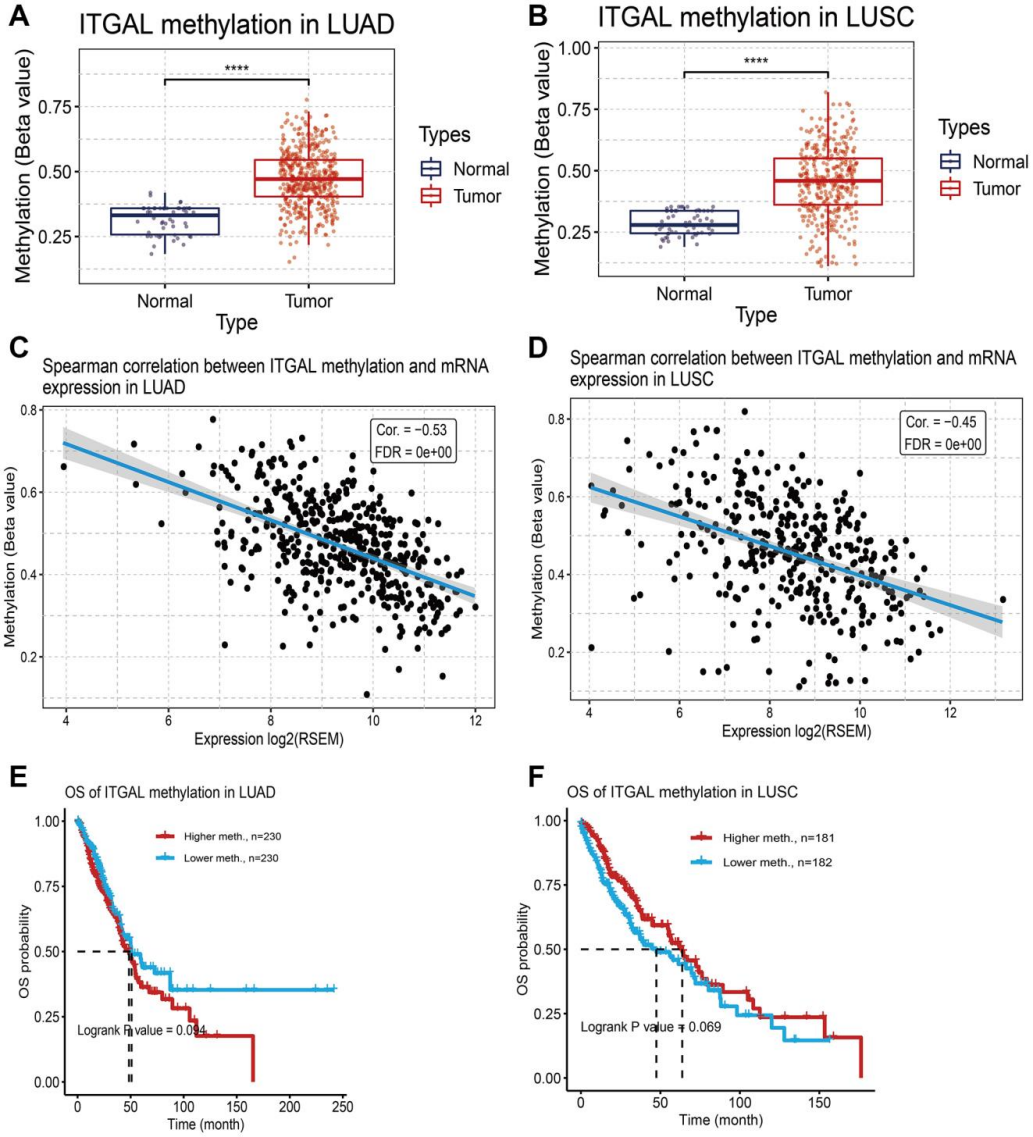
**DNA methylation analysis.** (**A**, **B**) The mean level of DNA methylation of ITGAL was significantly higher in NSCLC tissues than in normal tissues. (**C**, **D**) Regression analysis of the correlation between ITGAL expression and its DNA methylation status. (**E**, **F**) Prognosis of ITGAL DNA methylation in lung cancer patients. ^***^p < 0.001.

Linkedomics was used to get the genes that are positive or negative with ITGAL in NSCLC (Figure 4A, 4B). GO enrichment results confirmed that ITGAL was major involved in the immune response-related signaling pathway (Figure 4C). KEGG results indicated that ITGAL mainly participated in the T cell receptor pathway and Th17 cell differentiation (Figure 4D). DNA methylation plays an indispensable role in regulating cancer malignant progression and recurrence [19]. Hypermethylation of gene promoter regions usually results in gene expression decreased in tumors. We analysis of the correlations between DNA methylation and ITGAL in NSCLC. Results confirmed that DNA methylation of ITGAL was higher in NSCLC tissues than in normal tissues (Figure 5A, 5B). We also found a significant negative correlation between ITGAL levels and DNA methylation status (Figure 5C, 5D). However, the DNA methylation level of the ITGAL gene did not affect the prognosis of lung cancer patients (Figure 5E, 5F).

### Correlation of ITGAL expression and immune infiltrates

**Figure 6:**
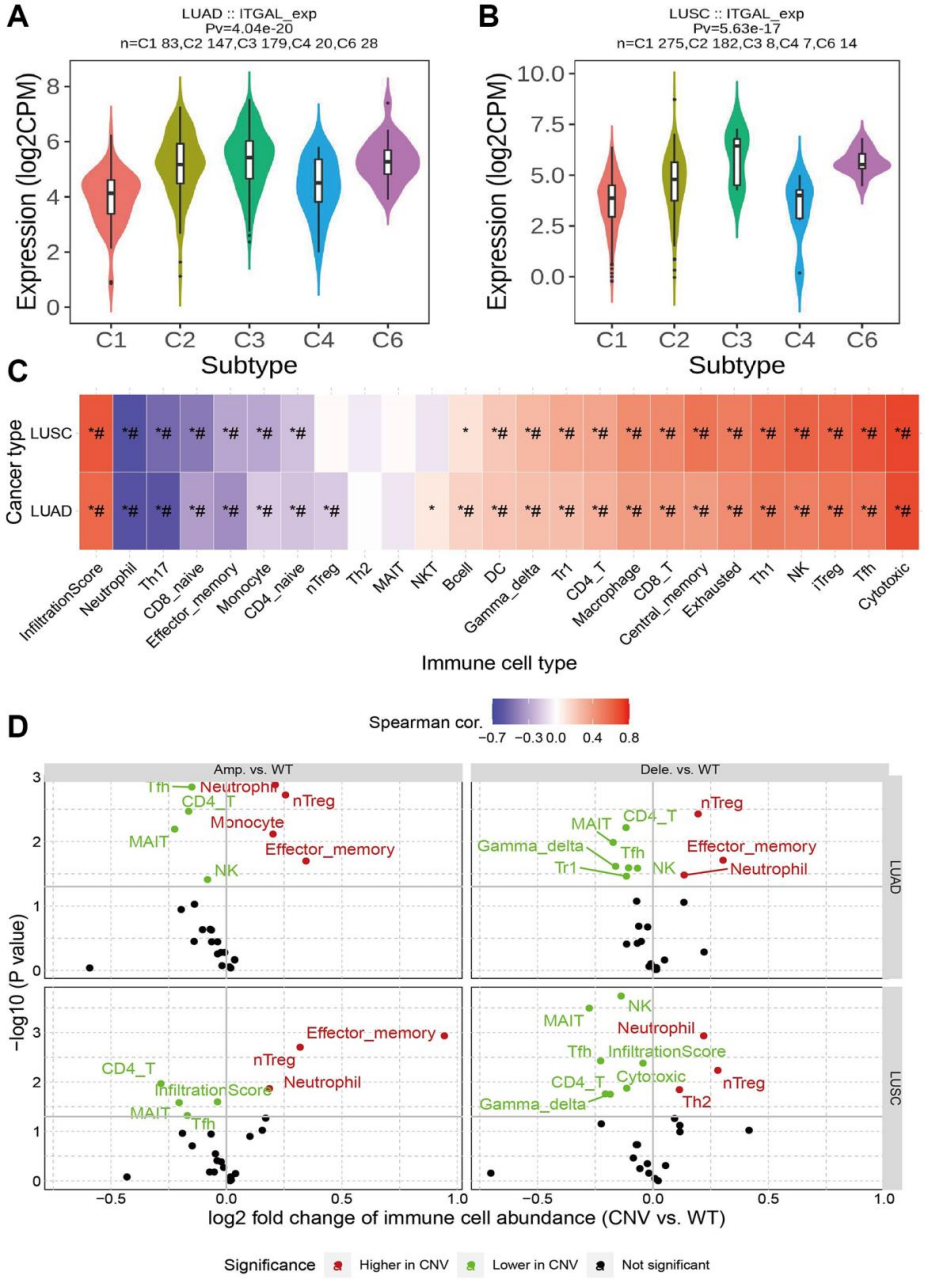
**The expression of the ITGALin immune subtype.** (**A**, **B**) The expression of ITGALin immune subtype of NSCLC. (**C**, **D**) Relation between ITGAL CNV and immune cell in NSCLC. ^*^p < 0.05.

**Figure 7:**
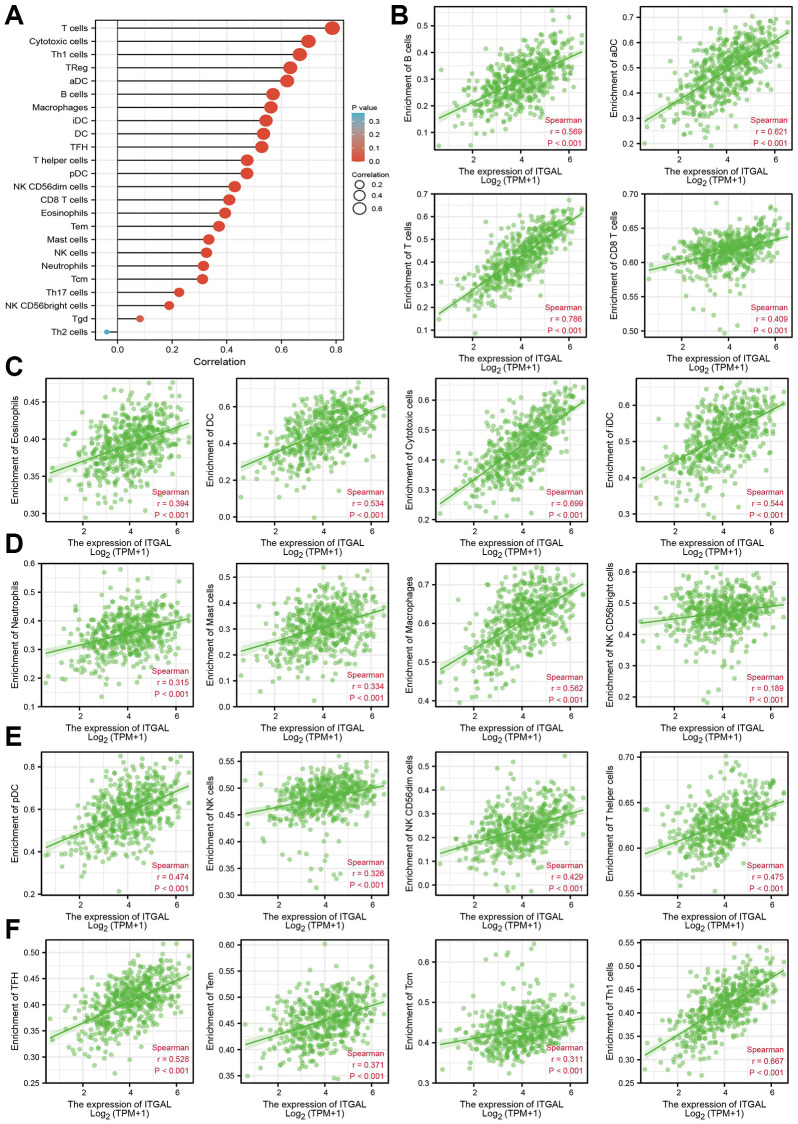
**Correlation between ITGAL expression and immune infiltrates.** (**A**–**F**) Correlation between ITGAL expression and diverse immune infiltrates in NSCLC.

**Figure 8:**
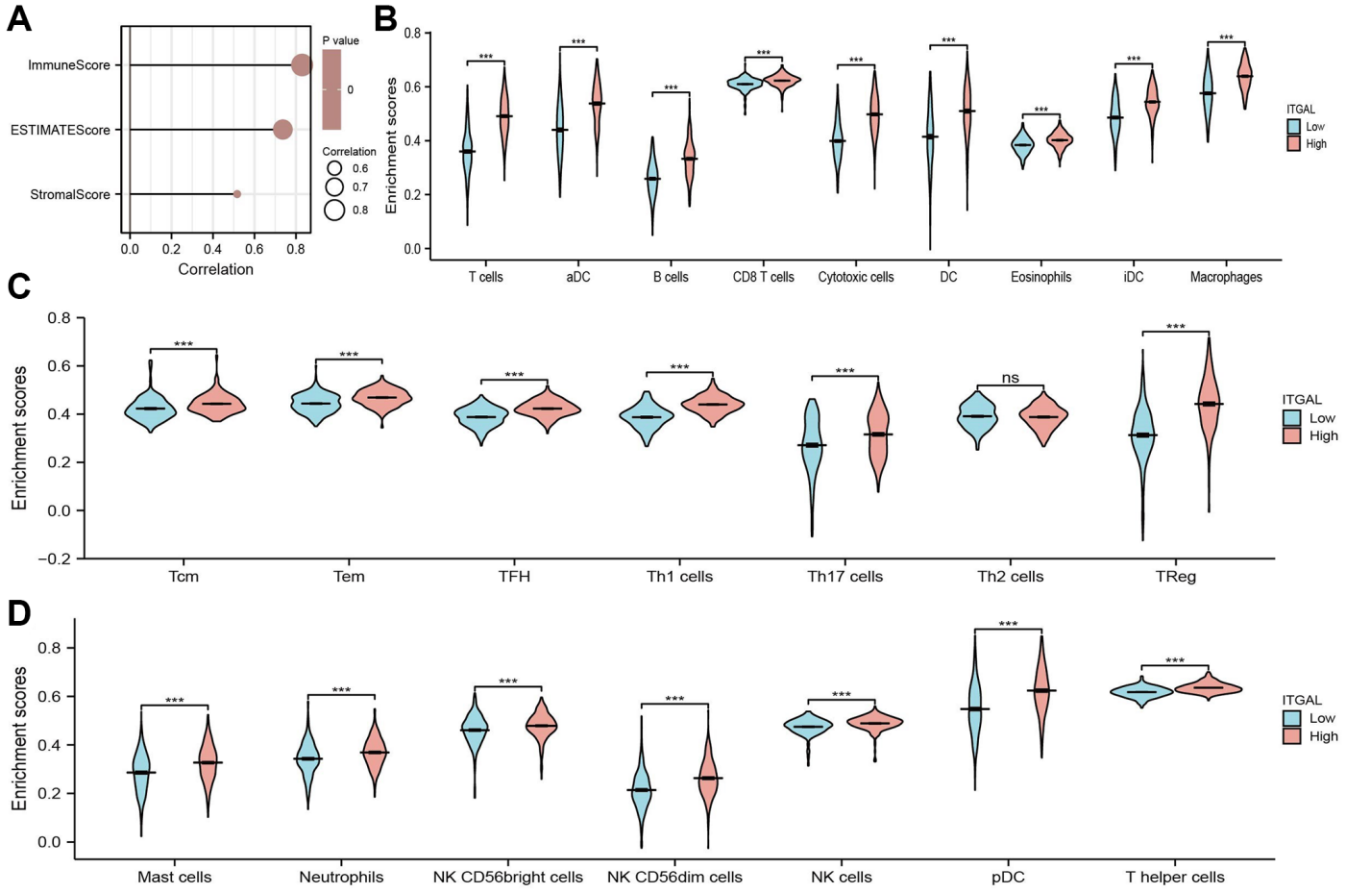
**Correlation between ITGAL expression and immune infiltrates.** (**A**) ITGAL expression was positively correlated with NSCLC immune scores, stromal scores, and ESTIMATE scores (**B**–**D**) The abundance of immune infiltrates of diverse immune cells based on the ITGAL high or low expression group. NS > 0.05 and ^***^p < 0.001.

We further explore the relationship between ITGAL levels and the tumor immune microenvironment of NSCLC. First, we found that ITGAL was highly expressed in the C3 immune subtype (Figure 6A, 6B). Further study indicated that there is a positive correlation between ITGAL CNV and cytotoxic, Treg, NK, Th1, Exhausted, Central-memory, CD8-T, Macrophage, and CD4-T cells in NSCLC (Figure 6C, 6D). Next, we confirmed that ITGAL was positively related to the 23 types of immune cells (Figure 7A–7F), immune scores, stromal scores, and ESTIMATE scores in NSCLC (Figures 8A). Finally, we showed that in the ITGA-high expression group, the infiltration level of most immune cells significantly increased (Figure 8B–8D).

**Figure 9:**
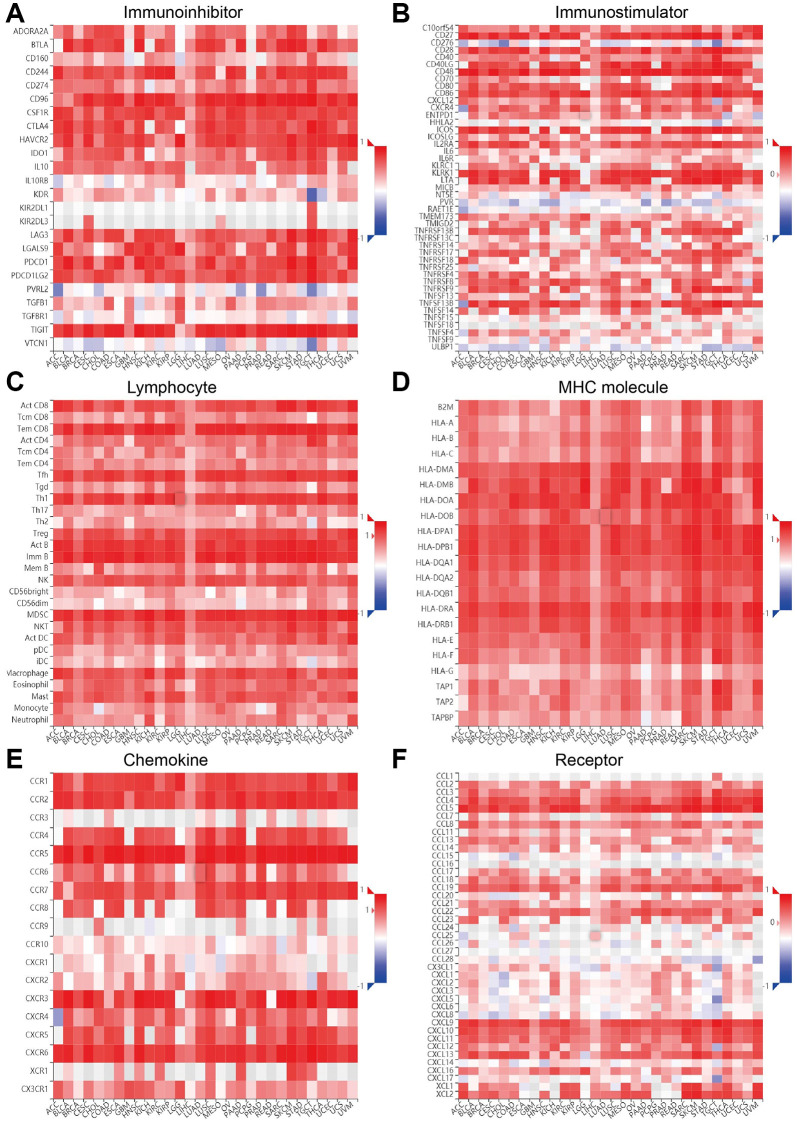
**Correlation between ITGAL expression and immune modulator.** (**A**–**F**) ITGAL expression was significantly positively related to the immune modulator, including the tumor-infiltrating lymphocytes (TILs), immune inhibitor, immunostimulator, MHC molecule, and chemokines as well as its receptors.

Immune checkpoint-related genes play an indispensable role in tumor progression and immune cell function. To further clarify the function of ITGAL in the immune microenvironment, we uncover that ITGAL level was positively related to the immune modulator, including the tumor-infiltrating lymphocytes (TILs), immune inhibitor, immunostimulator, MHC molecule, chemokines as well as its receptors (Figure 9A–9F).

### ITGAL-related ceRNA network in NSCLC

**Figure 10:**
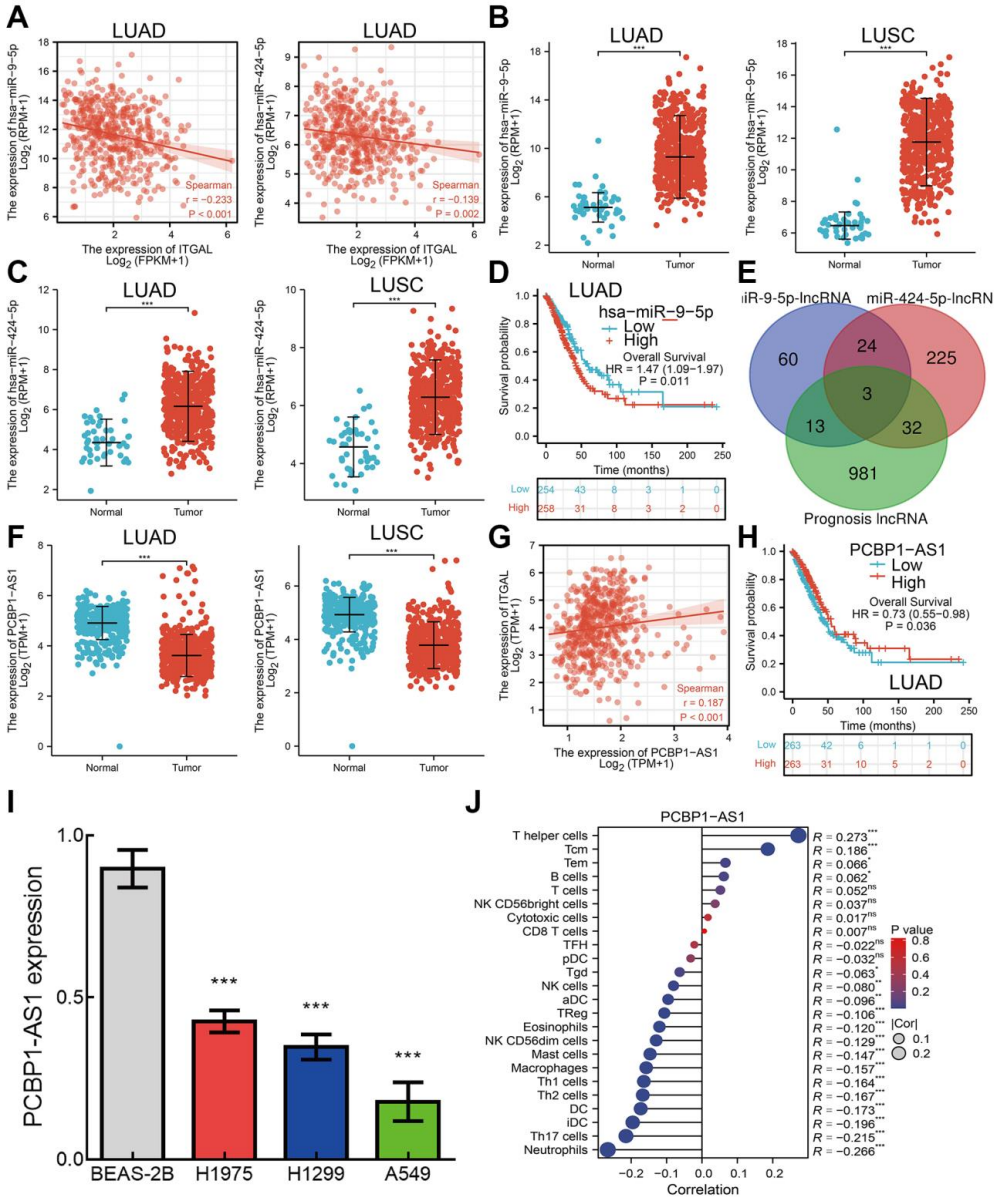
**ceRNA network of ITGAL in NSCLC.** (**A**) Correlations between miRNA and ITGAL in NSCLC. (**B**, **C**) Expression of miR-9-5p and miR-424-4p in NSCLC. (**D**) Prognosis of miR-9-5p in NSCLC. (**E**) Predicted the potential lncRNAs of miRNAs in NSCLC. (**F**) Expression of lncRNA in NSCLC. (**G**) Correlations between PCBP1-A1 and ITGAL in NSCLC. (**H**) Prognosis of PCBP1-A1 in NSCLC. (**I**) The RNA expression of PCBP1-A1 in lung cancer cell lines examined by qPCR assay, Actin as an internal reference gene. (**J**) Correlation between PCBP1-A1 expression and immune infiltrates. ^***^p < 0.001.

Studies showed that LncRNA is crucial for regulating gene expression at the post-transcriptional level [17]. To explore the upstream lncRNA/ceRNA network of ITGAL. We used starBase for prediction of potential miRNAs that bind with ITGAL. We obtained the 6 miRNAs in total, and according to the negative correlation with ITGAL expression in NSCLC, we identified 2 miRNAs, miR-9-5p and miR-424-5 (Figure 10A). Two miRNAs were downregulated in NSCLC and displayed worse patient prognosis (Figure 10B–10D).

In addition, we determine the lncRNAs that regulate miR-9-5p and miR-424-5 expression. By integrated analysis of two miRNA binding lncRNAs and lncRNAs with low expression in NSCLC and significant correlation with prognosis. We obtained 3 lncRNAs (Figure 10E), but only lncRNA PCBP1-A1 was downregulated in NSCLC and positively correlated with ITGAL expression in NSCLC (Figure 10F, 10G). More importantly, we uncover that lower lncRNA PCBP1-A1 expression correlated with adverse clinical outcomes in NSCLC patients (Figure 10H). More importantly, compared to normal lung epithelial cells, the expression level of PCBP1-A1 in lung cancer tumor cell lines is abnormally decreased (Figure 10I). Unlike ITGAL, PCBP1-A1 level is positively correlated with T helper cell infiltration (Figure 10J). These findings indicated that lncRNA PCBP1-AS1 is an upstream lncRNA for the miR-9-5p and miR-424-5/ ITGAL axis in NSCLC.

### Prognostic of ITGAL expressions in NSCLC based on immune cells

**Figure 11:**
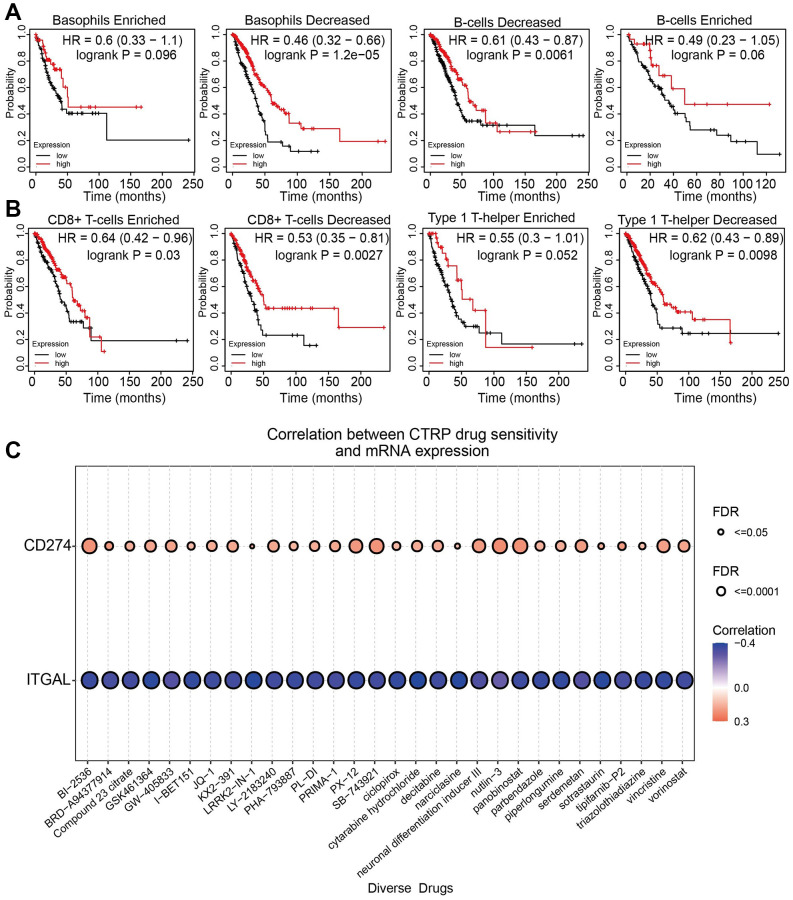
**Comparison of Kaplan-Meier survival curves of the high and low expression of ITGAL in NSCLC according to diverse immune cells.** (**A**, **B**) Overall survival curves of the higher and lower expression of ITGAL in NSCLC based on immune cell subgroups. (**C**) Correlations between ITGAL expression and sensitivity of various drugs by CTRP database.

Due to the significant correlation between ITGAL and infiltration of different immune cells, we further used Kaplan-Meier plotter analyses and found that low ITGAL levels in NSCLC in decreased Basophils cohort had a worse prognosis (Figure 11A, 11B). We also examined the relationship between ITGAL expression and diverse drug sensitivity in the cancer therapeutics response portal database. We showed that ITGAL expression was negatively correlated with the sensitivity of SNX-2112, GSK-J4, Cytarabine hydrochloride, Narciclasine, LRRK2-IN-1, Teniposide, Sotrastaurin, GSK461364, BRD-K66453893, Piperlongumine AT13387, I-BET151, Ciclopirox, CR-1-31B, Vincristine, Albendazole, PX-12, Belinostat, SR-II-138A, CHM-1, Isoliquiritigenin, Triazolothiadiazine and BI-2536 (r < −4, *p* < 0.001) (Figure 11C). This evidence suggested that ITGAL may regulate the sensitivity of the above different drugs and promote NSCLC progress. It is necessary to employ these drugs to verify their functions at the cellular level.

## DISCUSSION

ITGAL is a member of the integrin family which the dysregulation and is correlated with cancer progression and immune response. In this finding, we uncover that ITGAL expression was decreased in NSCLC, compared to normal lung tissues. Meanwhile, low ITGAL expression is associated with cancer stage, age, and overall event in NSCLC. This evidence suggests that ITGAL plays a crucial role in the progression of NSCLC. Previous studies showed that ITGAL was elevated in glioma and knockdown of ITGAL inhibited glioma cell growth [13]. ITGAL is also considered a very important prognostic marker in gastric cancer and acute myeloid leukemia [12, 20]. In our study, we found that ITGAL was downregulated in NSCLC cell lines.

Immunotherapy has made significant advances in the treatment of NSCLC while improving treatment efficacy through combination strategies has become a major direction in the field. ITGAL was previously reported to correlate with immune infiltrates in gastric cancer and acute myeloid leukemia [12, 20]. In this finding, we confirmed that ITGAL was positively related to the 23 types of immune cells, immune scores, stromal scores, and ESTIMATE scores in NSCLC.

The immunosuppressive microenvironment is thought to be one of the reasons for the poor response rate to immunotherapy. Interestingly, we found that ITGAL expression was significantly positively related to the tumor-infiltrating lymphocytes (TILs), immune inhibitor, immunostimulator, MHC molecule, chemokines as well as its receptors. These results indicated that ITGAL participated in the regulation of tumor immune cells. For the potential function and mechanism of ITGAL in NSCLC. Our results show that ITGAL is major involved in the NF-κ B signaling pathway. DNA methylation and lncRNA play an essential role in regulating gene expression [21]. In this manuscript, we found that the DNA methylation level of the promoter of ITGAL was lower in NSCLC than in normal lung tissues. We uncover that ITGAL expression was strongly correlated with DNA methylation in NSCLC.

To explore the mechanism of abnormally low expression of ITGAL in lung cancer, we used multiple comprehensive analyses and successfully found that lncRNA PCBP1-A1 may be down-regulated ITGAL expression via inhibited the expression of miR-9-5p and miR-424-5. Cancer is resistant to many traditional chemotherapy drugs, and analyzing and studying the molecular mechanisms behind tumor cell resistance and exploring potential drug resistance biomarkers is crucial for cancer treatment. In this study, we comprehensively analyzed the sensitivity between ITGAL expression and different drugs, providing the relevant basis for further pharmacological analysis in the future.
